# A Three-Year Follow-Up Study of Antibiotic and Metal Residues, Antibiotic Resistance and Resistance Genes, Focusing on Kshipra—A River Associated with Holy Religious Mass-Bathing in India: Protocol Paper

**DOI:** 10.3390/ijerph14060574

**Published:** 2017-05-29

**Authors:** Vishal Diwan, Manju Purohit, Salesh Chandran, Vivek Parashar, Harshada Shah, Vijay K. Mahadik, Cecilia Stålsby Lundborg, Ashok J. Tamhankar

**Affiliations:** 1 Department of Public Health and Environment, R.D. Gardi Medical College, Ujjain 456006, India; vivekstream@yahoo.co.in; 2 Department of Public Health Sciences, Global Health, Health Systems and Policy (HSP): Medicines focusing on antibiotics, Karolinska Institutet, Stockholm 17177, Sweden; manjuraj.purohit64@gmail.com (M.P.); saleshp@gmail.com (S.C.); cecilia.stalsby.lundborg@ki.se (C.S.L.); ejetee@gmail.com (A.J.T.); 3 International Centre for Health Research, Ujjain Charitable Trust Hospital and Research Centre, Ujjain 456001, India; 4 Department of Pathology, R.D. Gardi Medical College, Ujjain 456006, India; 5 Department of Microbiology, R.D. Gardi Medical College, Ujjain 456003, India; sharshada1955@yahoo.com; 6 R.D. Gardi Medical College, Ujjain 456006, India; uctharc@sancharnet.in; 7 Indian Initiative for Management of Antibiotic Resistance, Department of Environmental Medicine, R.D. Gardi Medical College, Ujjain 456006, India

**Keywords:** *Escherichia coli*, mass-bathing, antibiotic resistance, antibiotic resistance genes, water-quality, India, river, metals, seasons

## Abstract

*Background*: Antibiotic resistance (ABR) is one of the major health emergencies for global society. Little is known about the ABR of environmental bacteria and therefore it is important to understand ABR reservoirs in the environment and their potential impact on health. *Method/Design*: Quantitative and qualitative data will be collected during a 3-year follow-up study of a river associated with religious mass-bathing in Central India. Surface-water and sediment samples will be collected from seven locations at regular intervals for 3 years during religious mass-bathing and in absence of it to monitor water-quality, antibiotic residues, resistant bacteria, antibiotic resistance genes and metals. Approval has been obtained from the Ethics Committee of R.D. Gardi Medical College, Ujjain, India (No. 2013/07/17-311). *Results*: The results will address the issue of antibiotic residues and antibiotic resistance with a focus on a river environment in India within a typical socio-behavioural context of religious mass-bathing. It will enhance our understanding about the relationship between antibiotic residue levels, water-quality, heavy metals and antibiotic resistance patterns in *Escherichia coli* isolated from river-water and sediment, and seasonal differences that are associated with religious mass-bathing. We will also document, identify and clarify the genetic differences/similarities relating to phenotypic antibiotic resistance in bacteria in rivers during religious mass-bathing or during periods when there is no mass-bathing.

## 1. Background

Antibiotics enter the aquatic ecosystem either through the incomplete metabolism of used antibiotics or through the disposal of unused antibiotics [1,2,3]. More specifically, antibiotics enter the aquatic environment through direct discharge of wastewater from treatment plants, landfill leachate, runoff from agricultural and animal farms, leaking sewers, manure storage tanks or lagoons [4,5,6]. Alternatively, the aquatic environment can also receive antibiotics from spills or discharges during the manufacture and atmospheric dispersal of feed and manure dust [7].

The available literature has documented the presence of antibiotic residues in varying concentrations in aquatic environments, including river ecosystems [8,9,10,11,12,13]. It is further reported that antibiotics may be present in river water and other aquatic environments at levels that could not only alter the ecology of the environment, but also give rise to antibiotic resistant bacteria and genes that code for resistance [14,15]. Further, the presence of antibiotic resistant bacteria and genes in aquatic environments has become a serious concern for global public health [16,17,18,19]. Although antibiotics are produced naturally by autochthonous microbial communities, anthropogenic sources of antibiotics are considered to be a major driver of clinically important resistance phenotypes and genotypes [20]. It is also reported that selection of resistant bacteria can also occurs when antibiotic concentration are below minimal inhibitory concentration (MIC) [20,21]. In addition, the presence of heavy metals and genes that code for metal resistance in the river water interplay greatly with antibiotic resistance genes [22,23,24,25,26,27].

Further, in many low- and middle-income settings, a significant amount of sewage is not treated before its release into rivers or other environments [28]. Additionally, due to limited sanitary facilities in these settings, antibiotic resistant bacteria and genes along with human faecal material can spread into the environment [29]. *Escherichia coli* is abundantly found in the intestines of humans and animals, as well as in the environment, and thus it serves as a marker for the transfer of resistance from animal to human intestinal micro-flora [30]. There have been reports of seasonal- and temporal-variations in the concentration of antibiotic residues with changes in water-quality parameters and a variation in terms of the presence of antibiotic resistant bacteria and their associated genes [11,31,32,33,34,35].

Rivers are one of the important constituents of the environment and are used for many different purposes such as transport, irrigation, drinking, tourism and aquaculture. In some settings, rivers are considered holy and are used for religious activities including mass-gatherings [36] of religious importance of various sizes. Mass-bathing in rivers (a “holy dip”) on a large or small scale is a regular feature of Indian religious mass-gathering occasions. During holy dips, people may also drink the river water. These mass-bathing events typically last from 1 to 2 days on occasions such as a solar eclipse or new moon day, but can last for more than 1 month during the “Simhastha or Khumbh” Festival [37]. During mass-gatherings, the temporal and spatial, as well as the unique socioeconomic characteristics of the participants (i.e., specific events might attract participants in particular risk groups, thus increasing their chances of being a source of or becoming susceptible to infection) compound routine disease factors, such as the susceptibility to and effectiveness of transmission, leading to the emergence of infectious diseases and creating challenges for the prevention and control of these diseases [38,39,40].

To the best of our knowledge, there have been no long-term comprehensive studies that have followed antibiotic resistance and antibiotic residues in rivers associated with religious activities. Our research team has taken the lead in initiating a multi-disciplinary 3-year study to monitor water-quality, antibiotic and heavy metal residues, resistant bacteria and antibiotic resistance genes in *E. coli* at various strategic locations along the Kshipra River in Central India at regular intervals and also during special mass-gathering events. Such a comprehensive study will be the first of its kind and will provide data to inform future meaningful interventions in the area of effective environmental antibiotic stewardship. The study has the following objectives:

(1) To determine, over a 3-year period, water-quality, antibiotic residue levels, heavy metal residue levels, the coliform and *E. coli* burden and antibiotic resistance patterns in *E. coli* isolated from river-water and sediment.
(2) To determine, over a 3-year period, water-quality, antibiotic residue levels, heavy metal, residue levels, the coliform and *E. coli* burden, and antibiotic resistance patterns in *E. coli* isolated from river water and sediment during special mass-bathing occasions.
(3) To study the association between water-quality, antibiotic residues, heavy metal residues, the coliform burden and antibiotic resistance patterns of *E. coli* isolated from river-water and sediment over the 3-year study period.
(4) To study the association between water-quality, antibiotic residues, heavy metal residues, the coliform burden and antibiotic resistance patterns in *E. coli* isolated from river-water and sediment during special mass-bathing occasions.
(5) To evaluate the genomic commonality and diversity and pan-genomic correlates of antibiotic resistance genes of *E. coli* isolated from riverwater and sediment during different seasons and during special mass-bathing occasions.
(6) To explore the perceptions, beliefs and opinions of stakeholders and policy makers regarding antibiotic resistance, antibiotic residues and antibiotic use in the context of environmental pollution.

## 2. Methods

### 2.1. Design

This is a protocol for an environmental follow-up study with data collection over a 3-year period. Both quantitative and qualitative methods will be used.

### 2.2. Setting

The study will be conducted in Ujjain District of the Indian state of Madhya Pradesh (MP). MP is the second largest state in India with almost 77% of its 72 million population living in rural areas [41] with a low Human Development Index (HDI) score of 0.45 for India [42]. 

**Figure 1:**
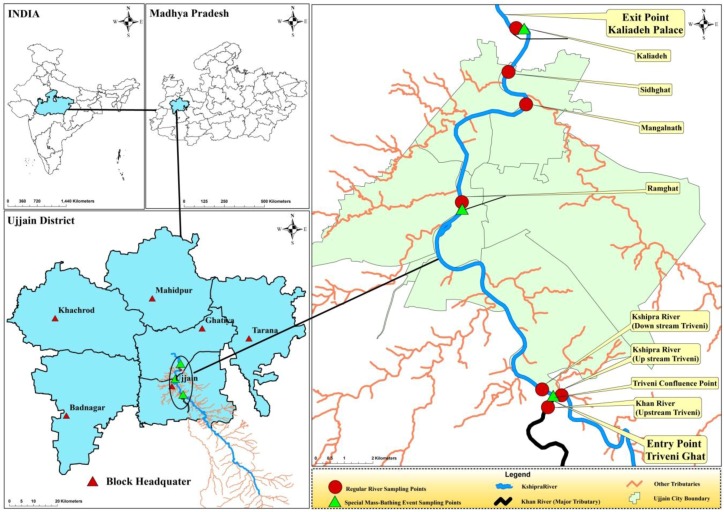
Geographical location of the sites for regular and mass-bathing sampling. The map shows (clockwise) India, Madhya Pradesh, Ujjain District and the sampling points for the Kshipra River, respectively.

Specifically, the study will be conducted within the reaches of the Kshipra River that flows through the city of Ujjain. The reach extends from Triveni Ghat to Kaliyadeh Palace, the respective entry and exit points of the river for the city of Ujjain (Figure 1).

### 2.3. About the Kshipra River

The Kshipra River is 195-km long, of which 93 km flows through Ujjain District. It originates in the Kokri Bardi Hills (747-m high). After crossing a 70-km pathway, it enters Ujjain District. Large quantities of pollutants enter the river from its main tributaries, especially from the River Khan (just upstream of Ujjain), which is the main source of industrial pollution in Kshipra [43]. Being a religious river, several small mass-gatherings are organised on the banks of the Kshipra River throughout the year. Additionally, Ujjain is one of the four cities in India that hosts one of the biggest religious mass-gatherings (KhumbhMela or Simhastha) every 12 years, an event where a massive temporary city is constructed to accommodate millions of worshipers seeking to bathe in the sacred water of the Kshipra River [40]. These mass-gathering and mass-bathing events around the banks of the river are observed by pilgrims and can be for a single day or last for several days.

### 2.4. Weather

The study area has four distinct seasons: summer, monsoon, post-monsoon and winter. The summer season (March to June) has average maximum temperatures ranging between 35 °C and 45 °C, with minimal rainfall, while the monsoon season (late June to September) witnesses an average of 172 mm of rainfall, with average maximum temperatures ranging between 29 °C and 31 °C. The post-monsoon season and the winter season (November to February) have average maximum temperatures ranging between 27 °C and 31 °C with limited rainfall [44].

### 2.5. Sampling

The study will involve the collection of river-water and sediment samples at regular interval is named as “regular river sampling” and also during special mass-bathing events named as “mass-bathing sampling” over a 3-year period. We will collect water and sediment samples at the same time of day during each sampling occasion.

#### 2.5.1. Selection of Sampling Points

A field survey was conducted to determine the sampling sites along the length of the selected reach of the Kshipra River. The criteria for the sampling locations include both the point and non-point sources of pollution, and the locations were chosen at places which were either mass-bathing spots or spiritually important places where the pollution load is expected to be very high. The sampling locations for “regular river sampling” and for mass-bathing events are described in detail in the subsequent sections.

#### 2.5.2. Sampling Points for “Regular River Sampling”

Seven major points in the selected reach running through Ujjain were identified along the river where sampling is to be conducted (Figure 1). Samples are to be collected for a total of 12 time-points from each of the seven locations: four times a year over a 3-year period, with each collection round occurring at an interval of 3 months, in the winter, summer, monsoon and post-monsoon seasons, to record changes over time and to determine any potential seasonal effects.

#### 2.5.3. Sampling Points for “Mass-Bathing” Events

Three major sampling points in Ujjain were identified along the river at which sampling for mass-bathing is proposed (Figure 1). During the study period of 3 years, 12 samplings will be done during mass-bathing events.

### 2.6. Sample Collection

#### 2.6.1. For “Regular River Sampling”

Water samples in duplicates from seven selected points will be collected from the left bank, the right bank and the centre of the river, and a composite of the three will be prepared by mixing the samples in equal quantities. Similarly soft-bottom sediment samples will be collected from all seven points using locally made Ekman Dredge sediment sampler.

#### 2.6.2. For “Mass-Bathing Events”

Duplicate samples from three selected points will be collected. The same sampling procedure as described earlierwill be applied. Samples (water and sediment) will be collected from each selected sampling point one day prior to a mass-bathing event, during the day of the mass-bathing event and 6 h–7 h after the mass-bathing event. 

### 2.7. Transport of Water and Sediment Samples to the Laboratory

In total, a 3.2 L water sample will be collected from each sampling point: 1 L for the analysis of antibiotic residues, 2 L for other water-quality estimations and 200 mL for colony counts and antibacterial-susceptibility tests. In addition, 2 kg of sediment will be collected from each sampling point: 1.5 kg for the analysis of antibiotic residues, 450 g for the sediment parameters and 50 g for colony counts and antibacterial-susceptibility tests. Samples for antibiotic residues will be transported to the laboratories of the Shriram Institute for Industrial Research (SIIR) in New Delhi within 12 h of sample collection in screw-capped amber bottles wrapped in silver foil [9]. Samples for studying the water-quality parameters and antibiotic susceptibility will be collected in plastic cans and sterilised 200-mL glass bottles, respectively. Sediment samples for colony counts and antibiotic susceptibility and for other parameters will be collected in 50-g sterilised conical-bottomed tubes and sterile 1 L plastic containers, respectively. The collected samples will be stored in an icebox during transportation to the laboratories on the day of collection. The cold chain will be maintained to keep the temperature at 4 °C. Colony counts, antibiotic susceptibility and water-quality analysis will be undertaken in the Central Research Laboratory of the R.D. Gardi Medical College, Ujjain. In the chemistry laboratory at New Delhi, the samples will be kept at −20 °C until analysis, while in the RDGMC Central Research Laboratory, the analysis will commence within 4 h of sample receipt.

### 2.8. Physical and Chemical Examination of Collected Water and Sediment Samples

#### 2.8.1. Water-Parameter Examination at Field Level

Immediately after the collection of the river water, each of the following parameters will be analysed at the respective sampling locations: ambient and water temperature, pH, conductivity, total dissolved solids (TDS), free carbon dioxide (free CO_2_), carbonate alkalinity and dissolved oxygen (DO). The ambient and water temperature will be measured using a mercury (Hg) thermometer graduated up to 100 °C with an accuracy of 0.1 °C–0.2 °C. The pH, conductivity and TDS will be measured using an ECO Tester hand-held digital pH meter (Tharmo Fisher Scientific, Mumbai, India), a 611-El Digital Conductivity Meter (Electronic India, Parwanoo, India) and a digital TDS meter (AM-TDS-01, Aquasol Digital, Rakiro Biotech Systems PVT LTD, Navi Mumbai, India) respectively. The titration method will be used to measure DO, free CO_2_ and carbonate alkalinity as per standard methods [45]. Flow will be monitored through an Environmental Measurement and Control (EMCON, Cochin, India) flow meter and the results will be reported as cm/s.

#### 2.8.2. Water- and Sediment-Parameter Examination in the Laboratory

At the RDGMC Central Research Laboratory in Ujjain, the collected samples will be analysed for turbidity, hardness, chloride, alkalinity, nitrate nitrogen, available phosphorous, total suspended solids (TSS), biochemical oxygen demand (BOD), chemical oxygen demand (COD) and total phosphorous.

A titration method will be used to measure chloride, bicarbonate alkalinity, total hardness and calcium hardness. TSS will be measured by filtering a known quantity of sampled water through a pre-weighed filter and then weighing the filter paper after drying at 105 °C. The 5-d BOD at 20 °C will be measured. The open reflex method will be applied to measure COD, while nitrate nitrogen, total phosphorus and the available phosphorus will be determined by spectrophotometer (UV-1800 Shimadzu, Kyoto, Japan). The parameters will be measured by applying the standard methods [46]. Turbidity will be determined by the Nephelo Turbidity Meter (Deluxe turbidity meter-385, Electronic India, Prwanoo, India). All sediment analysis of the various parameters will be carried out in the laboratory. The collected sediment samples will first be air-dried at room temperature before any analysis. Sediment samples will be analysed for pH, chloride, bicarbonates, nitrate nitrogen, available phosphorus and organic matter, as per standard methods [47].

#### 2.8.3. Analysis of Heavy and Other Metals

Water and sediment samples will be analysed for mercury, chromium, nickel, cobalt, manganese, cadmium, zinc, arsenic, sulphur and iron. An atomic mass spectrophotometer will be used for analysis.

### 2.9. Antibiotic Residue Analysis

Six antibiotics belonging to four major antibiotic groups—cephalosporins (ceftriaxone), fluoroquinolones (ofloxacin, ciprofloxacin, norfloxacin), sulfonamides (sulfamethoxazole) and imidazoles (metronidazole)—have been selected for residue analyses in the water and sediment samples. Total residual antibiotics as beta-lactam will also be analysed. The selection process was based on: (1) antibiotic residues found in the same geographical area in our previous studies [8], (2) the degree of antibiotic metabolism by the human body, (3) environmental stability, and (4) the known and suspected environmental impact of an antibiotic [48].

The determination of antibiotic residues in the collected river-water and sediment samples will be carried out by using solid-phase extraction followed by liquid chromatography tandem-mass spectrometry (LC-MS/MS) (Waters 2695 Series Alliance Quaternary Liquid Chromatography System, Waters Milford, MA, USA) with a triple quadruple mass spectrometer (Quatro-micro API, Micromass, Manchester, UK) equipped with an electro-spray interface and the Micromass Masslynx 4.1 software for data acquisition and processing. The methods used here are the same as those described in detail in our previous studies [8,9].

### 2.10. Microbiological Methods

The received samples will be processed as follows: (1)Ten-fold serial dilutions (1:100, 1:1000 as per turbidity of sample) of surface water will be made in 0.9% sterile saline (NaCl) solution. The whole sediment sample will be added to 100 mL of 0.9% normal saline and then serial ten-fold dilutions will be carried out. The diluted samples will be filtered following a standard membrane-filtration technique using nylon membrane filters of 47 mm in diameter with a pore size 0.45 µm. After filtration, the membrane will be taken out from the assembly and placed on selective and differential media for the identification and isolation of *E. coli* and non-*E. coli* isolates using HiCrome coliform agar for 24 h of incubation at 37 °C. (2) Bacterial enumeration will be carried out to estimate the total coliform count and total *E. coli* count in colony-forming units (CFUs) per 100 mL on agar. (3) The isolation and subsequent DNA extraction of six *E. coli* isolates per surface water and sediment sample will be undertaken for polymerase chain reaction (PCR) testing. (4) Antibiotic-susceptibility testing will simultaneously be performed using the Kirby Bauer disc-diffusion test on Muller Hinton (MH) agar for eight different classes of antibiotics using a bacterial suspension with 0.5 McFarland turbidity. The zone diameter of bacterial growth inhibition will be measured and interpreted as per Clinical and Laboratory Standard Institute (CLSI) guidelines [49].

To categorise the *E. coli* as resistant or sensitive to mercury (Hg), each strain will be tested on nutrient agar (HiMedia Laboratories, Mumbai, India) supplemented with 5 μM of Hg^2+^. If bacterial growth is present following incubation for 24 h–48 h at 37 °C, then this will be considered as an Hg-resistant *E. coli* strain. *E. coli* ATCC 35218 (Hg resistant) and *E. coli* ATCC 23724 (Hg susceptible) will be used as the control strains. The absence of growth will indicate the Hg susceptibility of the strain. These tests will be carried out in duplicate.

### 2.11. Molecular Methods

#### PCR Amplification of Genes

Various genes coding for β-lactamase such as CTX-M, TEM and SHV; plasmid-mediated quinolone-resistance genes—*qnrA*, *qnrB*, *qnrS*, *aac(6′)-Ib-cr* and *qepA*—and the carbapenem-resistance coding genes, OXA-48, IMP, VIM and NDM, will be amplified and identified with previously described primers [50] for *E. coli* isolated from all sediment and water samples. The phylogenetic grouping of all *E. coli* isolates will then be undertaken to group them as A, B1, B2 or D based on the *chuA*, *yjaA* and *TspE4C2* genes. These genes will be amplified by multiplex PCR, as mentioned previously [51]. *E. coli* possess metal-resistance genes that code for mercury these will be detected by PCR. Specific PCR primer sets will be used for the genes *mer A* that code for mercury resistance, [52,53]. All the amplified PCR products will be visualised using a gel documentation system.

The genomic and plasmid DNA will be extracted and purified using standard procedures from *E. coli* isolates which showed resistance to the selected antibiotics. DNA samples from all resistant isolates and from 20% of susceptible isolates will be subjected to whole-genome sequencing (WGS). The sequences will be assembled and analysed using the available *E. coli* reference genome [54,55].

### 2.12. Qualitative Study

We will conduct qualitative individual interviews and focus-group discussions (FGDs) to explore the knowledge, understanding and perceptions about antibiotic use, residues and resistance and its consequences on the environment and public health amongst various stakeholders (please refer to the Supplementary Material).

### 2.13. Data Management and Analyses

#### 2.13.1. Data Management

A specific database will be prepared for recording of the quantitative data. Bar codes will allow for the unique identification of each sample and will be used to keep track of samples both between the field and the laboratory and within the laboratory. Data entry will be done by trained entry operators. Senior researchers will supervise data entry. All data entered will be double checked for quality assurance. For FGDs and interviews, the transcripts and recorded material will be stored in a secure location. The identity of all interviewees will be removed from the transcripts before analysis to ensure confidentiality.

#### 2.13.2. Data Analysis for Quantitative Data

All quantitative data will be analysed using appropriate statistical methods. The descriptive analysis of the results from regular river sampling and special mass-bathing sampling will help us to better understand the distribution of various physical, chemical, microbiological and molecular variables collected over the 3-year study period. The season and type of sampling (special mass-bathing and regular river sampling) comparisons will be performed using either Chi-square tests or the Student’s *t*-test depending on the variable type. Furthermore, the differences in the outcome variables will be studied using appropriate statistical methods and a variety of regression models. The water-quality parameters, antibiotic residues, heavy and others metals, colony counts, antibiotic susceptibility and PCR results will be analysed for both regular and special mass-bathing events, both separately and in combination. An analysis of water quality and its effect on antibiotic resistance will be undertaken. The correlation, if any, between antibiotic residues in the surface-water and sediment samples with antibiotic resistant bacteria will be determined.

The generated genotypic data for regular river and special mass-bathing events will also be analysed to: (1) identify the genotypic antibiotic resistance patterns in the *E. coli* isolates with reference to chromosomal- and plasmid-mediated resistance using specific markers of plasmid-mediated resistance in *E. coli*; (2) identify and confirm the *E. coli* isolates present in the river-water and sediment samples (using a species-specific primer set/gene target); (3) identify and correlate the phylogenetic lineage of the antibiotic resistant *E. coli* isolates and group them according to the identified phylogenetic groups. This will identify the predominant circulating strains of specific phylogenetic group in the study setting (4) to allow us to follow, compare and correlate the coliform diversity pattern, resistance pattern and genetic pattern in isolates from regular sampling and special mass-bathing events.

#### 2.13.3. Data Analysis for Qualitative Data

Manifest and latent content analyses will be used for analyses of the qualitative data [56] (please refer to Supplementary Material).

### 2.14. Pilot Study

Several small-scale studies were conducted prior to the main study with the purpose of training research assistants in field and laboratory work as well as testing and validating the sampling and analysis methods. Minor changes in the data-collection procedures and methods were made in light of the pilot study.

## 3. Discussion

This protocol is for a unique study that addresses the issue of antibiotic residues and antibiotic resistance with a focus on the river environment in India with a typical socio-behavioural context of religious mass bathing. To understand the diverse and complex problem of antibiotic resistance, such a focus is important for the planning of context-appropriate interventions for the containment of antibiotic resistance.

There is sufficient information available and extensive research has been conducted in clinical settings regarding antibiotic resistance, however studies are less common when it comes to antibiotic resistance in the environment [57,58]. We know little about the antibiotic resistome of environmental bacteria and therefore it is important to understand antibiotic resistance reservoirs in the environment and their potential impact on human health. Additionally, current strategies and interventions mainly focus on risk assessment in clinical settings while non-clinical environments are largely ignored. In the context of the global increase in antibiotic resistance, attention also needs to be given to documenting the emergence and propagation of antibiotic resistant bacteria and genes in the environment and to understanding the relevant stakeholders’ perceptions, knowledge and beliefs regarding this problem [59,60,61].

We have previously reported antibiotic residues and resistance in hospital-associated waters in the same geographical location (Ujjain) by using concurrent continuous and grab sampling methods [9]. Seasonal- and temporal- variations in antibiotic residues and resistant bacteria were also reported by our research team [8]. We also estimated approximate quantities of major antibiotics released by hospitals into the environment. The wastewater from these studied hospitals ends up in the river that will be examined in this proposed work (the Kshipra River) and the outcomes of these studies can be correlated with the results of our earlier hospital studies.

Further, we have also reported a high level of antibiotic use and antibiotic resistance in *E. coli* as well as genetic determinants of the resistance and phylogenetic groupings of the resistant *E. coli* isolates in hospital wastewater [50] and stool samples in healthy children [62] (Commensal *E. coli* isolates from the stool samples of healthy children in the rural community in the same geographical area revealed the carriage of resistance to more than one antibiotic and co-resistance of cephalosporins and quinolones in 26% and 44% of the participants, respectively [63]. Furthermore, the extended-spectrum beta-lactamase (ESBL)-encoding genes *bla*CTX-M-15 and *bla*TEM-1 and plasmid-mediated quinolone-resistance *aac (6’)-Ib-cr*, *qnrA*, *qnrB* and *qepA* genes were detected in hospital-associated waters [50,64]. All these are also likely to end up in the river through the runoff of water, as sewage-treatment facilities are lacking in the region. This same research group has also found high antibiotic resistance in cow stools in rural areas of eastern India [65]. Earlier qualitative studies also found that farmers thought that antibiotic resistance and antibiotic use on farms must be influencing antibiotic resistance in general and antibiotic resistance in humans in particular [66]. Presence of antibiotic resistant bacteria has been detected in drinking water sources of rural areas. It can be speculated that this can be due to surface-water runoff from human/animal faecal sources [62].

A major strength of this protocol is the involvement of multi-disciplinary research members from India and Sweden with backgrounds in limnology, environmental medicine, agriculture, microbiology, molecular genetics, public health, epidemiology and pharmacology. Members of the research team have been working jointly for the past 10 years and have published numerous articles on antibiotic resistance and related areas. The same research group is also involved in a 2-year comprehensive study in which humans, animals and their natural ecosystems are being studied in relation to the health-seeking of health providers and caregivers, and to the socio-behavioural aspect of antibiotic use and environmental aspects of antibiotic resistance under the framework of “One Health” [67].

Our results on the seasonal and temporal dynamics of antibiotic residues, water-quality, coliform burden, metal residues and the antibiotic susceptibility patterns of regular river and special mass-bathing, when analysed collectively and separately will be useful in designing interventions to reduce antibiotic resistance and antibiotic residues in rivers, including those associated with mass-bathing. Further, results from the molecular studies will be helpful in understanding the diversity of relevant resistance genes and mobile genetic elements in the environment. Extended genome data from the sampled bacteria in the river environment during different seasons and with different dynamics during mass-bathing and regular flow will be beneficial in tackling the particulars of antibiotic resistance. The mapping of antibiotic resistancegenes nucleotide sequences of bacteria from an environmental origin will improve our understanding of antibiotic resistance ecology and will help to reduce the dissemination of clinically-relevant antibiotic resistant bacteria and the use of antibiotics (Antibiotic Stewardship Programs).

### Ethics Approval and Ethical Considerations

The study was approved by the Ethics Committee of the R.D. Gardi Medical College, Ujjain, MP, India (No: 2013/07/17-311). Water and sediment samples will be taken only from public land and no specific permission is required for this purpose. For qualitative studies oral and written information will be given. The aim of the study will be explained to the participants and information will be given that confidentiality will be guaranteed from the researchers and a consent form will be signed. Recording permission will be obtained from study participants for Interviews and focus group discussions.

## 4. Conclusions

Antibiotic resistance is a global public health challenge, especially in countries with fragmented health systems. With a limited focus on the environment, this problem is becoming increasingly difficult to address. We aimed to monitor water-quality, antibiotic and heavy metal residues, resistant bacteria and antibiotic resistance genes in *E.coli* at various strategic locations along the Kshipra River in Central India associated with religious mass bathing. The research team holds the view that by employing qualitative, quantitative, microbiological and molecular genetics methods, the results will help in developing future interventions in resource-constrained settings to contain antibiotic resistance and expenditure on infectious-disease management.

## Availability of Data and Materials

Due to ethical and legal restrictions, all inquiries should be made to The Chairman, Ethics Committee, R.D. Gardi Medical College, Agar Road, Ujjain, India 456006 (E-mails: iecrdgmc@yahoo.in; uctharc@bsnl.in), giving full details of the intended use. Upon verification of genuineness of the inquiry, the data that will be generated from various studies of this protocol will be made available. For reference, please quote ethical permission no. No: 2013/07/17-311.

## Supplementary Materials

The following are available online at www.mdpi.com/1660-4601/14/6/574/s1.
