# Supplementary material for: A Three-Year Follow-Up Study of Antibiotic and Metal Residues, Antibiotic Resistance and Resistance Genes, Focusing on Kshipra—A River Associated with Holy Religious Mass-Bathing in India: Protocol Paper
Source: Int J Environ Res Public Health. 2017 May 29;14(6):574. doi: 10.3390/ijerph14060574 (PMC5486260; doi:10.3390/ijerph14060574)
Supplement: Supplementary file 1 [file ijerph-14-00574-s001.pdf]

Supplementary Material-1

Qualitative Study

We will conduct qualitative individual interviews and focus-group discussions (FGDs) to explore the knowledge, understanding and perceptions about antibiotic use, residues and resistance and its consequences on the environment and public health amongst various stakeholders. We will approach officials from national, state and regional health departments, environment pollution departments, agriculture and animal husbandry departments, intellectuals and academics, political and religious leaders, drug and pharmacy dispensers, local farmers, poultry farm owners and peoples engaged in mass-bathing events for individual interviews. The interviews will be conducted to explore what approaches stakeholders take in regards to the growing problem of antibiotic resistance in non-clinical/environmental contexts. We will further explore their detailed views on the possible consequences of and solutions for antibiotic residues and resistance in terms of public health and the health system. Further, we will conduct FGDs with the group of stakeholders mentioned above. The FGDs will focus on the views and opinions of the various stakeholders in regards to resistance and its relation to the environment and broader public health. For both individual interviews and FGDs, relevant topic guides with open-ended questions and suitable probing questions will be prepared. Interviews and FGDs will be conducted in English or Hindi, depending on the language that is most suitable for the participant(s). All individual interviews and FGDs, if permitted by participants, will be audio-recorded and will then be transcribed and translated into English for analysis.

Data Analysis for Qualitative Data

Manifest and latent content analyses will be used for analyses of the qualitative data. Meaning units will be extracted from the transcripts. A meaning unit is part of the original transcript that carries a specific meaning, and generally consists of several words taken directly from the transcript. The meaning units will be then condensed to condensed meaning units and will further condensed into codes. Similar codes will be grouped into sub-categories and will further grouped into categories and ultimately the underlying themes will identified. More than two co-authors will perform the coding independently. No pre-defined coding scheme will be used. The analysis will be performed manually. The results will be repeatedly discussed among the authors from India and Sweden with backgrounds in limnology, environmental medicine, agriculture, microbiology, molecular genetics, public health, epidemiology and pharmacology.
